# The Relative Validity and Reproducibility of Food Frequency Questionnaires in the China Kadoorie Biobank Study

**DOI:** 10.3390/nu14040794

**Published:** 2022-02-14

**Authors:** Chenxi Qin, Yu Guo, Pei Pei, Huaidong Du, Ling Yang, Yiping Chen, Xi Shen, Zumin Shi, Lu Qi, Junshi Chen, Zhengming Chen, Canqing Yu, Jun Lv, Liming Li

**Affiliations:** 1 Department of Epidemiology and Biostatistics, School of Public Health, Peking University Health Science Center, Beijing 100191, China; chenxi.qin@ki.se (C.Q.); lmleeph@vip.163.com (L.L.); 2 National Coordinating Center of China Kadoorie Biobank, Chinese Academy of Medical Sciences, Beijing 102308, China; guoyu@kscdc.net (Y.G.); peipei@kscdc.net (P.P.); 3 National Clinical Research Center for Cardiovascular Diseases, Fuwai Hospital, Chinese Academy of Medical Sciences, Beijing 102308, China; 4 Clinical Trial Service Unit and Epidemiological Studies Unit (CTSU), Nuffield Department of Population Health, University of Oxford, Oxford OX3 7LF, UK; huaidong.du@ndph.ox.ac.uk (H.D.); ling.yang@ndph.ox.ac.uk (L.Y.); yiping.chen@ndph.ox.ac.uk (Y.C.); zhengming.chen@ndph.ox.ac.uk (Z.C.); 5 CKB Office, Suzhou Centers for Disease Control and Prevention, Suzhou 215004, China; baixi1224@163.com; 6 Department of Human Nutrition, College of Health Sciences, QU Health, Qatar University, Doha 2713, Qatar; zumin@qu.edu.qa; 7 Department of Epidemiology, School of Public Health and Tropical Medicine, Tulane University, New Orleans, LA 70112, USA; lqi1@tulane.edu; 8 NHC Key Laboratory of Food Safety Risk Assessment, China National Center for Food Safety Risk Assessment, Beijing 100022, China; chenjunshi@cfsa.net.cn; 9 Peking University Center for Public Health and Epidemic Preparedness & Response, Beijing 100191, China; 10 Key Laboratory of Molecular Cardiovascular Sciences, Peking University, Ministry of Education, Beijing 100191, China

**Keywords:** food frequency questionnaire, validity, reproducibility

## Abstract

Background: Short versions of qualitative and quantitative food frequency questionnaires (FFQs) are widely used to assess usual food intake. However, fewer studies evaluated their relative validity and reproducibility in the Chinese population. Methods: This study compared 12-day 24-h dietary recalls with qualitative and quantitative FFQs designed by the China Kadoorie Biobank (CKB) study to assess the relative validity. Two FFQs were administered in the second and third seasons and compared to evaluate the reproducibility. Statistical tests included Spearman correlation coefficients, weighted kappa, and cross-classification. Results: A total of 432 participants were eligible after stratifying by age, sex, and four regions. In the validation of qualitative FFQ, adjusted Spearman coefficients were between 0.23 and 0.59, and weighted kappa coefficients ranged from 0.61 to 0.88, except for fresh vegetables. The percentage of correct classification was highest in fresh vegetables and lowest in fresh fruit, but the percentages of extreme classification were below 3.0%. Corresponding Spearman and kappa coefficients for the reproducibility were 0.17–0.56 and 0.62–0.90. Furthermore, the correct classification constituted between 35.6 and 93.3% of all participants. Regarding the relative validity of the quantitative FFQ, Spearman coefficients ranged from 0.14 to 0.69 in addition to dried vegetables and carbonated soft drinks. For items with more than two-thirds of total participants consumed, weighted kappa coefficients were from 0.57 to 0.79; correct classification percentages were between 34.6% and 67.5%. Spearman and kappa coefficients for the reproducibility of the quantitative FFQ were 0.15–0.71 and 0.60–0.86, respectively; correct classification percentages varied from 47.8% to 71.6%. Conclusion: Most food items from the qualitative FFQ showed acceptable or even good relative validity and reproducibility in the CKB study. Likewise, major food items in the quantitative FFQ were valid and reproducible, but poor performances of dried vegetables and carbonated soft drinks indicated the need for modification and validation in future research.

## 1. Introduction

Diet acts as a pivotal modifiable risk factor in the progression of various chronic diseases. Dietary records, dietary recalls, and food frequency questionnaires (FFQs) are commonly used to assess dietary intake in population-based studies. The FFQ is the most time- and cost-effective way to assess long-term dietary intakes and widely administered in epidemiological studies [1]. FFQ includes qualitative and quantitative FFQs depending on whether to estimate amounts. Several previous studies showed that estimating food weights explained a limited percentage of between-person variation [2,3,4,5], but this would demand trained staff and time. Although food items in the FFQ should be informative as much as possible, researchers have to make compromises with reduced items considering research aims and respondent burden. It is notable that less detailed food items could lead to rough definitions and hereafter introduce bias from weight estimation [1]. Hence, studies should design an appropriate FFQ based on their purposes and resources. In addition, the validity and reproducibility of FFQ, especially a short one, is crucial for future analyses of dietary information. Lacking a gold standard, most validation studies used multiple dietary records or recalls as the optimal reference and summarised correlation coefficients between 0.4 and 0.6 for the quantitative FFQ and those between 0.2 and 0.5 for the qualitative FFQ [4].

Long FFQs have been used to measure nutrient levels in the Chinese population, such as the Chinese National Nutrition and Health Survey (149 food items) [6] and the Shanghai Women’s and Men’s Health Study (79 and 81 food items, respectively) [7,8]. However, large observational studies usually have limited resources to collect detailed dietary information and lesser needs to measure macronutrient and micronutrient levels [9,10]. For example, the China Kadoorie Biobank (CKB), which enrolled around half a million adults aged 30–79 years in 10 sites, administered a 12-item qualitative FFQ at baseline and a 20-item quantitative FFQ in the second resurvey to describe the long-term intake of common food groups [11,12]. In this context, a short FFQ with good validity and reproducibility is more realistic and practical, but there is scarce evidence about the short FFQ in the Chinese population [7,8,13]. Thus, this study aims to assess the relative validity and reproducibility of the short qualitative and quantitative FFQs in the CKB study, which other Chinese studies can adopt in the future.

## 2. Methods

### 2.1. FFQs in the CKB

The CKB study administered a qualitative FFQ at baseline (2004–2008) and the first resurvey (2008–2009) and then switched to a quantitative FFQ in the second resurvey (2013). 

The short qualitative FFQ chose 12 food items, including rice, wheat products, other staple foods (millet, corn, etc.), meat, poultry, fish/seafood, eggs, fresh vegetables, fresh fruit, dairy products, preserved vegetables, and dairy products according to recommendations from the Chinese Dietary Guidelines. Five frequency options were never or rarely, monthly, 1–3 days/week, 4–6 days/week, and daily. 

The quantitative FFQ retained the first nine food items in the qualitative FFQ and split the remaining three items into two or three subgroups (Supplementary Table S1). In addition, four new items were added, including pure fruit/vegetable juice, dried vegetables, carbonated soft drinks and other cold soft drinks. Alternative frequency levels remained the same as the qualitative FFQ. Participants estimated the average amount assisted by colour plates picturing the usual size and weight of food items.

### 2.2. Relative Validity and Reproducibility of FFQ 

Supplementary Figure S1 illustrates the field survey flow. Multiple 24-h dietary records or dietary recalls are widely used as the “gold” standard to assess the relative validity [1]. Considering that dietary records depend on the education level and compliance of participants, the present study took multiple 24-h dietary recalls (24 h DRs) as the reference. To avoid the bias caused by the seasonal food supply, dietary information was collected in four consecutive days from three seasons (summer, winter, and spring or autumn). Four investigation days included three workdays and one weekend day. The interval time between seasons was more than two months. Trained interviewers asked participants about all the foods they consumed and corresponding amounts during the past 24 h each day. For food recipes recorded in China Food Composition (2004 and 2009 editions) [14,15], participants estimated the overall weight; otherwise, participants reported each ingredient and its weight, except for condiments. 

In the reproducibility study, participants completed the first FFQ before 24 h DRs in the second season; in the third season, they answered the second FFQ after 24 h DRs. Colour plates from the second resurvey were provided as well. 

This study was approved by the Institutional Review Board of Peking University Health Science Center. All participants gave their written consent before joining the study.

### 2.3. Study Population

Considering the geographical location (urban/rural, southern/northern), food availability and dietary diversity in each site, the present study chose 13 villages or administrative communities from 4 out of 10 CKB study sites, including 1 urban site (Qingdao) and 3 rural sites (Zhejiang, Sichuan and Henan) to represent the CKB population. Eligible participants satisfied three criteria: (1) joining the baseline survey and the first and second resurveys; (2) aged less than 70 years old by 31 December 2016; (3) completing all questionnaires and signing the informed consent form. When multiple individuals fitted criteria in one household, one participant was randomly selected if they were of the same sex, otherwise, the male one was selected because there were fewer eligible male individuals. Among these candidates, the study randomly selected participants by sex and age groups (<50, 50–59, ≥60 years). Individuals with two circumstances were excluded: (1) unemployed and having more than half of lunches and suppers outside the home; (2) employed and having more than half of suppers outside because it was difficult to perform the face-to-face interview.

To validate the FFQ, 200–300 individuals are recommended for 3-day 24 h DRs and 100–200 individuals for 14–28 days of 24 h DRs [1]. After consultation with nutritional epidemiologists, the present study set the sample size at 480, taking a 20% loss follow-up rate into account. The field survey started in September 2015 and ended in August 2016. Finally, 432 participants were qualified for the qualitative FFQ and 416 for the quantitative FFQ after exclusion of those with an average daily energy intake outside of the 2–99 percentiles in the 24 h DRs.

### 2.4. Quality Control

After completing the field survey in each season, interviewers input questionnaires into a predesigned website and coded ingredients or recipes according to China Food Composition tables [14,15]. Ten percent of the overall questionnaires were randomly selected with stratification on survey sites and interviewers. Then, staff checked input errors and calculated percentages of missing, duplicate, and wrong items. If any percentage exceeded 1%, the corresponding interviewer examined all questionnaires he or she had completed. This process repeated until these indicators were lower than 1%. Finally, independent nutritional epidemiologists reviewed food codes.

### 2.5. Statistical Analyses

In FFQs, we assigned the midpoint value to each level (0, 0.5, 2, 5, and 7 days per week) and treated it as a continuous variable. Then, it was multiplied by the estimated amount and divided by seven was the average daily amount. In 24 h DRs, consuming a food item for 0, 1, 2–6, 7–10, 11–12 days corresponded to 5 frequency options in FFQs, respectively. The continuous frequency level (days per week) was the product of days that a participant consumed a specific food item and 7/12. The summing weight of a particular item divided by 12 generated the average daily amount, then it was categorized into three groups by tertiles. 

Percentages of frequency levels and median daily amounts were listed and compared between 24 h DRs and two FFQs using Wilcoxon tests. Cross-classification (percentages classified into the same, adjacent and extreme groups) and weighted kappa statistics were used to test the agreement at the group level [16]. The performance is good if more than 50% of the respondents were correctly classified and less than 10% were grossly classified; while it is considered to be bad if the correct classification percentage is below 50% and the extreme classification percentage exceeds 10% [16,17]. The weight for kappa was defined as 1 if frequency levels were in the same group, 0.5 if they were in adjacent groups, and 0 if they were in extreme groups [17]. A kappa value ≥0.61 represents a good outcome, 0.20–0.60 represents an acceptable one, and <0.20 means a poor one, respectively [16]. Age-, sex-, and region-adjusted Spearman coefficients were calculated to examine the strength and direction of the association at the individual level due to skewed distribution of data. The average daily energy intake derived from 24 h DRs was additionally adjusted when evaluating the relative validity of the qualitative FFQ. The Spearman coefficient greater than or equal to 0.50, between 0.20 and 0.49, and less than 0.20 indicate good, acceptable, and poor outcomes, respectively [16].

## 3. Results

A total of 432 participants completed all surveys. About 49.8% were men, 22.5% were urban residents, and the mean age was 55.0 years (standard deviation: 7.7 years) (Table 1). The median interval time between seasons was 3.3 months (interquartile: 3.0–4.7 months). 

### 3.1. Relative Validity and Reproducibility of the Qualitative FFQ

**Figure 1:**
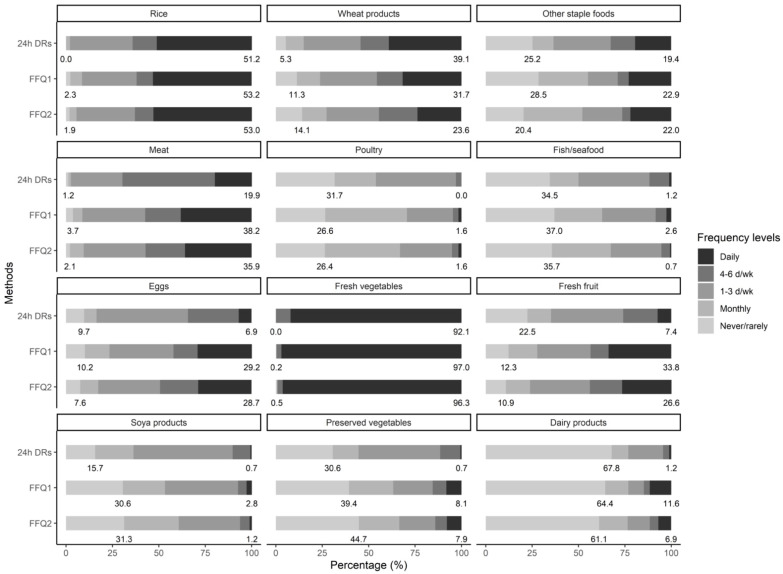
Percentages of frequency levels in 12-day 24 h DRs and 2 qualitative FFQs. FFQ: food frequency questionnaire; 24 h DRs: 24-h dietary recalls. Numbers below each bar represent the percentage of non-consumption (**left**) and daily consumption (**right**), respectively.

Figure 1 illustrates percentages of five frequency levels in 24 h DRs and FFQs (Supplementary Table S2). Twenty-four-h DRs reported higher percentages of daily wheat consumption but lower percentages of daily meat, eggs, and fresh fruit consumption compared with two qualitative FFQs. Daily wheat and fresh fruit intakes were more common in the first FFQ than in the second FFQ. In particular, more than 95% of participants consumed fresh vegetables every day. In 24 h DRs, foods from the qualitative FFQ contributed 88.8% of average daily energy intake and those from the quantitative FFQ accounted for 89.1% of average daily energy intake (Supplementary Table S3). 

Comparisons between 24 h DRs and qualitative FFQs showed that 62.1% (preserved vegetables) to 99.6% (fresh vegetables) of participants were in the same or adjacent frequency levels (Table 2). In particular, 89.3% of respondents reported daily consumption of fresh vegetables in both methods. All percentages of extreme classification were below 2.2% (fresh fruit). Except for fresh vegetables, average weighted kappa coefficients ranged from 0.61 (meat) to 0.88 (rice), and Spearman coefficients were between 0.23 (other staple foods) and 0.59 (fish/seafood) after adjusting for age, sex, and region. Comparisons between each FFQ and 24 h DRs were listed in Supplementary Tables S4 and S5.

In the reproducibility study, individuals reporting the same frequency levels constituted about 35.6% (soya products) to 93.3% (fresh vegetables), and those choosing extreme frequency levels were highest in dairy products (5.3%) (Table 3). In addition to fresh vegetables, average weighted kappa coefficients ranged from 0.62 (poultry) to 0.90 (rice), and adjusted Spearman coefficients varied between 0.17 (soya products) and 0.56 (rice). 

### 3.2. Relative Validity and Reproducibility of the Quantitative FFQ

Quantitative FFQs demonstrated a higher intake of fresh and salted vegetables but a lower intake of wheat products, other staple foods, and soya products (excluding liquids) in comparison with 24 h DRs (Table 4). The median levels for most food items were approximate in two FFQs, except for eggs (15.7 g/d in the first FFQ vs. 31.4 g/d in the second FFQ).

Validity studies showed that average Spearman coefficients ranging from 0.14 (fresh vegetables) to 0.69 (pickled vegetables) after adjustment for age, sex, region and daily energy intake, but those of dried vegetables (0.04) and carbonated soft drinks (0.05) were insignificant (Table 5). For some food groups, cross-classification and weighted kappa statistics could not be calculated because more than two-thirds of respondents reported never or rare consumption in FFQs. Regarding the rest items, a range of 34.6% (dried vegetables) to 67.5% (rice) of participants were correctly classified into the same tertile, while those who were grossly misclassified into opposite tertiles varied from 0.7% (wheat products) to 23.6% (salted vegetables). Weighted kappa coefficients for these food items ranged between 0.57 for fresh vegetables and 0.79 for rice. Comparisons of each FFQ with 24 h DRs were in Supplementary Tables S6 and S7. 

Adjusted Spearman correlation coefficients to assess the reproducibility were from 0.15 (other staple foods) to 0.71 (pickled vegetables), except for dried vegetables (0.06, *p* < 0.05) and carbonated soft drinks (0.04, *p* < 0.05) (Table 6). Participants in the same tertile accounted for about 47.8% (dried vegetables) to 71.6% (rice), and those in opposite tertiles constituted between 0.2% (rice) and 29.1% (salted vegetables). The weighted kappa was highest in salted vegetables (0.86) and lowest in fresh vegetables (0.60).

## 4. Discussion

This study compared repeated short qualitative and quantitative FFQs of CKB to assess the reproducibility and used 12-day 24-h dietary recalls as the reference method to evaluate the relative validity. Numerous studies have assessed the relative validity and reproducibility of FFQs and suggested good performance with the correlation coefficient greater than 0.5 and acceptable performance with the coefficient between 0.20 and 0.49 [16,17,18]. Good performance was also implicated when the kappa statistic greater than 0.60 or extreme classification percentage below 10% and right classification percentage above 50% [16]. In the present study, the qualitative FFQ showed acceptable even good relative validity and reproducibility. In the quantitative FFQ, food items demonstrated acceptable validity and reproducibility except for dried vegetables, pure fruit/vegetable juice, carbonated soft drinks, and other soft drinks. 

Instead of measuring the favourable effects of particular nutrients, the purpose of the CKB baseline survey was to describe characteristics of habitual consumption [19], investigate disease risks contributed by certain food items or the overall dietary pattern [20,21], and avoid confounding bias due to diet. The short food list with broad definitions posed great challenges to weight estimation. Therefore, the CKB study only administered a qualitative FFQ. Later, the second resurvey used a quantitative FFQ among a randomly selected subpopulation aiming to estimate usual portion sizes for food groups at baseline [20,22]. 

The method to assess the validity and reproducibility in this study was in line with that of prior studies such as the Chinese National Nutrition and Health Survey, Shanghai Women’s and Men’s Health Study, European Prospective Investigation into Cancer and Nutrition, and UK Biobank [6,7,8,23,24]. The dietary record is usually recognized to be the “gold standard” to evaluate the validity, but it is more applicable in respondents with high motivation and literate ability. Hence, this study chose dietary recalls as the second optimal method such as in previous studies [7,25,26]. To minimize the recall bias, participants were encouraged to record foods and beverages according to the time. Participants were interviewed for 12 days (including working and weekend days) in three seasons to maximally address the influence of day-to-day variation and seasonality. When assessing the reproducibility, a longer interval between two FFQs could result in underestimation because of the long-term variation [27,28], but a shorter interval might lead to overestimation since individuals tend to remember the last answers. Two FFQs were 3.3 months apart that was in accordance with the recommendation for an FFQ collecting dietary habits in one year [1].

The quantitative CKB FFQ showed good or acceptable validity and reliability for nine overlapping food items in the qualitative and quantitative FFQs except for fresh vegetables. The consumption level of fresh vegetables might be still influenced by the diversity and accessibility across seasons, subsequently causing large variations in the amount. The acceptable performance of other staple foods resulted from the rough definition, which made it difficult to estimate the average amount for participants. The most probable explanation for the poor performance of dried vegetables was that the second resurvey did not clearly define the wet and dried weight. Poor results of carbonated and other soft drinks were because of infrequent consumption in the target population. Spearman coefficients for other groups were acceptable, but researchers need to be careful to interpret the results since more than two-thirds of total respondents did not consume these foods in the present study. 

In the qualitative FFQ, weighted kappa coefficients were greater than 0.60 and Spearman coefficients exceeded 0.2 in all food groups except for fresh vegetables. Although correct classification percentages accounted for less than 50% in most groups, a majority of respondents were classified into adjacent frequency groups, and misclassification percentages were still below 10%. This could result from five frequency levels in the FFQ, which was different from three or four groups in other studies when describing cross-classification [16]. Both the kappa and Spearman coefficients of fresh vegetables were insignificant, but this was caused by the high prevalence of daily consumption (>90%) [29]. High percentages of correct classification (about 90%) and low percentages of extreme classification (<1%) still indicated good validity and reproducibility. However, the limited discriminative ability of frequency levels for fresh vegetables can contribute little variation in future studies. This indicates that food groups with high-frequency intake need more precise assessments in the Chinese population, such as daily frequency, amount, or type of vegetables. 

The present study investigated multiple days of 24 h DRs, including weekdays and weekends in three seasons to minimize within-person variation and seasonal influences and capture the dietary habits throughout the year. We selected these four sites based on north–south and rural–urban dissimilarities, as well as their diet cultures to represent the CKB population to a great extent. A large sample size also increased the power compared with other studies [7,23,24]. Yet, several limitations should be acknowledged. Firstly, the validity and reproducibility of FFQs were usually assessed before administering in the target population. The CKB study originally focused on the disease risk associated with a variety of environmental factors, such as smoking and alcohol consumption, with adjustment for covariates such as dietary behaviours. A detailed evaluation of FFQs was indeed neglected in the first place. Still, the present study found good or acceptable outcomes for the major food items. In addition, the CKB study periodically performed resurveys and offered an opportunity to upgrade the FFQ with a better discriminative ability or comprehensive definitions for some items. Secondly, the great diversity in each food group impeded the calculation of nutrient levels and their associations with disease risks. Thirdly, respondents should be representative of the entire population. However, the CKB participants were geographically scattered, making stratified random sampling impractical [1]. This study has balanced the feasibility of field survey and representativeness as much as possible. 

## 5. Conclusions

In summary, the present study evaluated the relative validity and reproducibility of qualitative and quantitative FFQs administered in the CKB baseline and resurveys and found major food items with good or acceptable performance. However, foods such as dried vegetables and carbonated soft drinks are not suitable for further research. 

## Figures and Tables

**Table 1:** Age, sex, and region distribution among 432 participants.

Regions	Age Group	Qualitative FFQ			Quantitative FFQ		
		Men	Women	Overall	Men	Women	Overall
Qingdao (Urban)	<50	0	8	97	0	8	89
	50–59	12	10		9	9	
	≥60	29	38		29	34	
Sichuan (Rural)	<50	17	24	108	17	24	101
	50–59	18	17		18	14	
	≥60	19	13		19	9	
Henan (Rural)	<50	33	22	119	33	22	118
	50–59	17	19		17	18	
	≥60	13	15		13	15	
Zhejiang (Rural)	<50	19	16	108	19	16	108
	50–59	18	19		18	18	
	≥60	20	16		20	16	
Overall		215	217	432	212	203	416

FFQ: food frequency questionnaire.

**Table 2:** Average coefficients to compare the qualitative FFQ and 12-day 24 h DRs.

Food Groups	Weighted Kappa	Adjusted Spearman	Cross-Classification			
			Same Groups	Adjacent Groups	Extreme Groups	Others
Rice	0.88	0.54	76.0	19.1	0.2	4.8
Wheat products	0.80	0.37	46.2	35.1	<0.1	18.8
Other staple foods	0.80	0.23	39.7	40.2	0.9	19.3
Meat	0.61	0.34	41.3	49.3	<0.1	9.4
Poultry	0.64	0.25	43.7	44.7	<0.1	11.6
Fish/seafood	0.73	0.59	47.7	41.8	<0.1	10.4
Eggs	0.65	0.49	34.6	44.7	0.5	20.3
Fresh vegetables	0.06 *	0.02 *	89.3	10.1	0.4	0.3
Fresh fruit	0.72	0.53	31.4	41.3	2.2	25.2
Soya products	0.65	0.36	39.8	38.7	<0.1	21.6
Preserved vegetables	0.81	0.39	38.5	27.5	0.9	33.3
Dairy products	0.75	0.47	58.1	22.6	1.3	18.0

FFQ: food frequency questionnaire; 24 h DRs: 24-h dietary recalls. The weight for kappa was defined to be 1 if the frequency levels were in the same group, 0.5 if they were in adjacent groups, and 0 if they were in extreme groups. Spearman coefficients were adjusted for age, sex, and region. * Coefficients were not significant (*p* > 0.05).

**Table 3:** Coefficients to compare two qualitative FFQs.

Food Groups	Weighted Kappa	Adjusted Spearman	Cross-classification			
			Same Groups	Adjacent Groups	Extreme Groups	Others
Rice	0.90	0.56	75.9	17.1	0.7	6.3
Wheat products	0.81	0.43	46.5	37.3	<0.1	16.3
Other staple foods	0.85	0.28	47.7	32.2	2.3	17.8
Meat	0.77	0.36	49.3	33.8	0.7	16.2
Poultry	0.62	0.26	46.1	40.7	<0.1	13.2
Fish/seafood	0.75	0.49	53.2	34.3	0.5	12.1
Eggs	0.77	0.41	39.4	32.9	1.4	26.4
Fresh vegetables	−0.01 *	−0.03 *	93.3	5.3	0.7	0.7
Fresh fruit	0.81	0.42	41.2	30.1	3.5	25.2
Soya products	0.65	0.17	35.6	38.0	0.5	25.9
Preserved vegetables	0.75	0.31	39.4	32.6	2.5	25.5
Dairy products	0.82	0.39	57.4	22.2	5.3	15.1

FFQ: food frequency questionnaire. The weight for kappa was defined to be 1 if the frequency levels were in the same group, 0.5 if they were in adjacent groups, and 0 if they were in extreme groups. Spearman coefficients were adjusted for age, sex and region. * Coefficients were not significant (*p* > 0.05).

**Table 4:** Median daily levels of food groups from 12-day 24 h DRs and 2 quantitative FFQs.

Food Groups	Median (Interquartile) g/d			Wilcoxon Test 1st vs. 2nd FFQ
	24 h DRs	1st Quantitative FFQ	2nd Quantitative FFQ	
**Original groups**				
Rice	91.5 (46.3–199.9)	103.6 (28.6–300.0)	107.1 (39.3–250.0)	0.46
Wheat products	74.9 (11.9–194.4)	42.9 (23.2–107.1) *	42.9 (8.9–100.0) *	0.03 *
Other staple foods	24.8 (0.8–72.3)	10.7 (0.0–50.0) *	14.3 (7.1–50.0)	0.05 *
Meat	45.0 (25.6–67.7)	50.0 (28.6–100.0)	50.0 (28.6–100.0) *	0.12
Poultry	6.2 (0.0–16.6)	7.1 (0.0–14.3)	7.1 (0.0–28.6) *	0.08
Fish/seafood	8.3 (0.0–31.0)	7.1 (0.0–28.6)	7.1 (0.0–28.6)	0.84
Eggs	29.6 (13.3–55.0)	15.7 (15.7–55.0)	31.4 (15.7–55.0)	0.15
Fresh vegetables	33.3 (6.7–106.1)	57.1 (14.3–107.1) *	57.1 (28.6–142.9) *	0.63
Fresh fruit	228.3 (66.4–306.3)	200.0 (150.0–300.0) *	200.0 (150.0–300.0) *	0.29
**Split groups**				
Soya products (excluding liquids)	13.3 (4.2–28.8)	7.1 (0.0–28.6) *	7.1 (0.0–28.6) *	0.19
Soymilk	0.0 (0.0–0.0)	0.0 (0.0–0.0)	0.0 (0.0–0.0) *	0.28
Salted vegetables	4.2 (0.0–11.3)	3.6 (0.0–14.3) *	0.0 (0.0–3.6) *	<0.05 *
Pickled vegetables	0.0 (0.0–0.0)	0.0 (0.0–3.6) *	0.0 (0.0–0.0) *	0.51
Milk	0.0 (0.0–20.0)	0.0 (0.0–17.9)	0.0 (0.0–17.9)	0.49
Yoghurt	0.0 (0.0–0.0)	0.0 (0.0–0.0)	0.0 (0.0–0.0)	0.28
Other dairy foods	0.0 (0.0–0.0)	0.0 (0.0–0.0)	0.0 (0.0–0.0)	0.43
**Added groups**				
Dried vegetables	0.9 (0.0–2.8)	3.6 (0.0–7.1) *	3.6 (0.0–7.1) *	0.06
Pure fruit/vegetable juiceǂ	-	0.0 (0.0–0.0)	0.0 (0.0–0.0)	0.11
Carbonated soft drinks	0.0 (0.0–0.0)	0.0 (0.0–0.0) *	0.0 (0.0–0.0) *	0.14
Other cold soft drinks	0.0 (0.0–0.0)	0.0 (0.0–0.0)	0.0 (0.0–0.0) *	0.03 *

24 h DRs: 24-h dietary recalls; FFQ: food frequency questionnaire. Original groups refer to food items shared by the qualitative and quantitative FFQ. Split groups refer to food items in the qualitative FFQ but split into subgroups in the quantitative FFQ. Added groups refer to new food items in the quantitative FFQ. The weight for kappa was defined to be 1 if the frequency levels were in the same group, 0.5 if they were in adjacent groups, and 0 if they were in extreme groups. Spearman coefficients were adjusted for age, sex, and region. * Comparisons using the Wilcoxon test were significant (*p* < 0.05). ǂ No participants consumed pure fruit or vegetable juice in the 24 h DRs.

**Table 5:** Average coefficients to compare the quantitative FFQ and 12-day 24 h DRs.

Food Groups	Adjusted Spearman	Weighted Kappa	Cross-Classification		
			Same Tertile	Adjacent Tertile	Opposite Tertile
**Original groups**					
Rice	0.42	0.79	67.5	31.9	0.6
Wheat products	0.34	0.71	57.9	41.4	0.7
Other staple foods	0.15	0.71	54.1	38.7	7.2
Meat	0.32	0.68	47.9	39.1	13.1
Poultry	0.26	0.66	47.9	41.8	10.4
Fish/seafood	0.42	0.72	55.8	38.8	5.4
Eggs	0.41	0.69	52.0	39.2	8.9
Fresh vegetables	0.14	0.57	38.3	44.0	17.8
Fresh fruit	0.48	0.71	54.5	39.0	6.6
**Split groups**					
Soya products (excluding liquids)	0.27	0.63	44.2	42.6	13.2
Soymilk	0.27	-	-	-	-
Salted vegetables	0.30	0.81	54.0	22.5	23.6
Pickled vegetables	0.69	-	-	-	-
Milk	0.43	-	-	-	-
Yoghurt	0.36	-	-	-	-
Other dairy foods	0.31	-	-	-	-
**Added groups**					
Dried vegetables	0.04 *	-	36.2	41.5	22.4
Pure fruit/vegetable juiceǂ	-	-	-	-	-
Carbonated soft drinks	0.05 *	-	-	-	-
Other cold soft drinks	0.18	-	-	-	-

FFQ: food frequency questionnaire; 24 h DRs: 24-h dietary recalls. Original groups refer to food items shared by the qualitative and quantitative FFQ. Split groups refer to food items in the qualitative FFQ but split into subgroups in the quantitative FFQ. Added groups refer to new food items in the quantitative FFQ. The weight for kappa was defined to be 1 if the frequency levels were in the same group, 0.5 if they were in adjacent groups, and 0 if they were in extreme groups. Spearman coefficients were adjusted for age, sex, and region. The blank cell indicated the percentage of zero consumption exceeded 66.7%. * Coefficients were not significant (*p* > 0.05). ǂ No participant consumed pure fruit or vegetable juice in the 24 h DRs.

**Table 6:** Coefficients to compare the quantitative FFQs.

Food Groups	Adjusted Spearman	Weighted Kappa	Cross-Classification		
			Same Tertile	Adjacent Tertile	Opposite Tertile
**Original groups**					
Rice	0.40	0.79	71.6	28.1	0.2
Wheat products	0.31	0.75	58.9	39.4	1.7
Other staple foods	0.15	0.72	57.0	34.6	8.4
Meat	0.32	0.68	54.3	36.3	9.4
Poultry	0.21	0.65	50.7	36.1	13.2
Fish/seafood	0.39	0.71	55.3	36.8	7.9
Eggs	0.41	0.69	47.1	42.3	10.6
Fresh vegetables	0.16	0.60	45.0	40.9	14.2
Fresh fruit	0.50	0.75	49.8	37.5	12.7
**Split groups**					
Soya products (excluding liquids)	0.26	0.62	42.1	42.8	15.1
Soymilk	0.26	-	-	-	-
Salted vegetables	0.38	0.86	51.4	19.5	29.1
Pickled vegetables	0.71	-	-	-	-
Milk	0.38	-	-	-	-
Yoghurt	0.35	-	-	-	-
Other dairy foods	0.39	-	-	-	-
**Added groups**					
Dried vegetables	0.06 *	-	47.8	36.8	15.4
Pure fruit/vegetable juice	-	-	-	-	-
Carbonated soft drinks	0.04 *	-	-	-	-
Other cold soft drinks	0.22	-	-	-	-

FFQ: food frequency questionnaire. Original groups refer to food items shared by the qualitative and quantitative FFQ. Split groups refer to food items in the qualitative FFQ but split into subgroups in the quantitative FFQ. Added groups refer to new food items in the quantitative FFQ. The weight for kappa was defined to be 1 if the frequency levels were in the same group, 0.5 if they were in adjacent groups, and 0 if they were in extreme groups. Spearman coefficients were adjusted for age, sex, and region. The blank cell indicated the percentage of zero consumption exceeded 66.7%. * Coefficients were not significant (*p* > 0.05).

## Data Availability

The access policy and procedures are available at www.ckbiobank.org (accessed on 14 January 2022).

## Supplementary Materials

The following are available online at https://www.mdpi.com/article/10.3390/nu14040794/s1, Supplementary Table S1. Food items in the quantitative and qualitative FFQs in the China Kadoorie Biobank study; Supplementary Table S2. Percentages of frequency levels in 12-day 24 h DRs and two FFQs; Supplementary Table S3. Average daily intake energy of food groups in 24 h DRs; Supplementary Table S4. Coefficients to compare the first qualitative FFQ and 12-day 24 h DRs; Supplementary Table S5. Coefficients to compare the second qualitative FFQ and 24 h DRs; Supplementary Table S6. Coefficients to compare the first quantitative FFQ and 24 h DRs; Supplementary Table S7. Coefficients to compare the second quantitative FFQ and 24 h DRs; Supplementary Figure S1. The study design to assess the relative validity and reproducibility of qualitative and quantitative FFQs in the China Kadoorie Biobank study.

## References

[1] Willett W. (2012). Nutritional Epidemiology.

[2] Noethlings U., Hoffmann K., Bergmann M.M., Boeing H. (2003). European Investigation into C, Nutrition. Portion size adds limited information on variance in food intake of participants in the EPIC-Potsdam study. J. Nutr..

[3] Samet J.M., Humble C.G., Skipper B.E. (1984). Alternatives in the collection and analysis of food frequency interview data. Am. J. Epidemiol..

[4] Cade J., Thompson R., Burley V., Warm D. (2002). Development, validation and utilisation of food-frequency questionnaires—A review. Public Health Nutr..

[5] Hunter D.J., Sampson L., Stampfer M.J., Colditz G.A., Rosner B., Willett W.C. (1988). Variability in portion sizes of commonly consumed foods among a population of women in the United States. Am. J. Epidemiol..

[6] Zhao W.-H., Huang Z.-P., Zhang X., He L., Willett W., Wang J.-L., Hasegawa K., Chen J.-S. (2010). Reproducibility and Validity of a Chinese Food Frequency Questionnaire. Biomed. Environ. Sci..

[7] Shu X.O., Yang G., Jin F., Liu D., Kushi L., Wen W., Gao Y.-T., Zheng W. (2004). Validity and reproducibility of the food frequency questionnaire used in the Shanghai Women’s Health Study. Eur. J. Clin. Nutr..

[8] Villegas R., Yang G., Liu D., Xiang Y.-B., Cai H., Zheng W., Shu X.O. (2007). Validity and reproducibility of the food-frequency questionnaire used in the Shanghai men’s health study. Br. J. Nutr..

[9] Hu F.B., Satija A., Rimm E.B., Spiegelman D., Sampson L., Rosner B., Camargo C.A., Stampfer M., Willett W.C. (2016). Diet Assessment Methods in the Nurses’ Health Studies and Contribution to Evidence-Based Nutritional Policies and Guidelines. Am. J. Public Health.

[10] Bohlscheid-Thomas S., Hoting I., Boeing H., Wahrendorf J. (1997). Reproducibility and relative validity of energy and macronutrient intake of a food frequency questionnaire developed for the German part of the EPIC project. European Prospective Investigation into Cancer and Nutrition. Int. J. Epidemiol..

[11] Chen Z., Lee L., Chen J., Collins R., Wu F., Guo Y., Linksted P., Peto R. (2005). Cohort profile: The Kadoorie Study of Chronic Disease in China (KSCDC). Int. J. Epidemiol..

[12] Chen Z., Chen J., Collins R., Guo Y., Peto R., Wu F., Li L., on behalf of the China Kadoorie Biobank (CKB) Collaborative Group (2011). China Kadoorie Biobank of 0.5 million people: Survey methods, baseline characteristics and long-term follow-up. Int. J. Epidemiol..

[13] Zhang C.X., Ho S.C. (2009). Validity and reproducibility of a food frequency Questionnaire among Chinese women in Guangdong province. Asia Pac. J. Clin. Nutr..

[14] National Institute of Nutrition and Health, China CDC (2002). China Food Composition 2002.

[15] National Institute of Nutrition and Health, China CDC (2009). China Food Composition.

[16] Lombard M.J., Steyn N.P., Charlton K.E., Senekal M. (2015). Application and interpretation of multiple statistical tests to evaluate validity of dietary intake assessment methods. Nutr. J..

[17] Masson L.F., Mcneill G., Tomany J.O., Simpson J., Peace H., Wei L., Grubb D., Bolton-Smith C. (2003). Statistical approaches for assessing the relative validity of a food-frequency questionnaire: Use of correlation coefficients and the kappa statistic. Public Health Nutr..

[18] Cui Q., Xia Y., Wu Q., Chang Q., Niu K., Zhao Y. (2021). A meta-analysis of the reproducibility of food frequency questionnaires in nutritional epidemiological studies. Int. J. Behav. Nutr. Phys. Act..

[19] Qin C., Yu C., Du H., Guo Y., Bian Z., Lyu J., Zhou H., Tan Y., Chen J., Chen Z. (2015). Differences in diet intake frequency of adults: Findings from half a million people in 10 areas in China. Zhonghua Liu Xing Bing Xue Za Zhi.

[20] Qin C., Lv J., Guo Y., Bian Z., Si J., Yang L., Chen Y., Zhou Y., Zhang H., Liu J. (2018). Associations of egg consumption with cardiovascular disease in a cohort study of 0.5 million Chinese adults. Heart.

[21] Lv J., Yu C., Guo Y., Bian Z., Yang L., Chen Y., Tang X., Zhang W., Qian Y., Huang Y. (2017). Adherence to Healthy Lifestyle and Cardiovascular Diseases in the Chinese Population. J. Am. Coll. Cardiol..

[22] Du H., Li L., Bennett D., Guo Y., Key T.J., Bian Z., Sherliker P., Gao H., Chen Y., Yang L. (2016). Fresh Fruit Consumption and Major Cardiovascular Disease in China. N. Engl. J. Med..

[23] Kaaks R., Slimani N., Riboli E. (1997). Pilot phase studies on the accuracy of dietary intake measurements in the EPIC project: Overall evaluation of results. European Prospective Investigation into Cancer and Nutrition. Int. J. Epidemiol..

[24] Liu B., Young H., Crowe F.L., Benson V.S., Spencer E.A., Key T.J., Appleby P.N., Beral V. (2011). Development and evaluation of the Oxford WebQ, a low-cost, web-based method for assessment of previous 24 h dietary intakes in large-scale prospective studies. Public Health Nutr..

[25] Boeing H., Bohlscheid-Thomas S., Voss S., Schneeweiss S., Wahrendorf J. (1997). The relative validity of vitamin intakes derived from a food frequency questionnaire compared to 24-h recalls and biological measurements: Results from the EPIC pilot study in Germany. European Prospective Investigation into Cancer and Nutrition. Int. J. Epidemiol..

[26] Bradbury K.E., Young H.J., Guo W., Key T.J. (2018). Dietary assessment in UK Biobank: An evaluation of the performance of the touchscreen dietary questionnaire. J. Nutr. Sci..

[27] Goldbohm R.A., van’t Veer P., van den Brandt P.A., van’t Hof M.A., Brants H.A., Sturmans F., Hermus R.J. (1995). Reproducibility of a food frequency questionnaire and stability of dietary habits determined from five annually repeated measurements. Eur. J. Clin. Nutr..

[28] Tsubono Y., Nishino Y., Fukao A., Hisamichi S., Tsugane S. (1995). Temporal change in the reproducibility of a self-administered food frequency questionnaire. Am. J. Epidemiol..

[29] Sim J., Wright C.C. (2005). The kappa statistic in reliability studies: Use, interpretation, and sample size requirements. Phys. Ther..

