# Supplementary material for: The Relative Validity and Reproducibility of Food Frequency Questionnaires in the China Kadoorie Biobank Study
Source: Nutrients. 2022 Feb 14;14(4):794. doi: 10.3390/nu14040794 (PMC8879142; doi:10.3390/nu14040794)
Supplement: Supplementary file 1 [file nutrients-14-00794-s001.zip › nutrients-1576853-supplementary.pdf]

The relative validity and reproducibility of food frequency questionnaires in the China Kadoorie Biobank Study

Supplementary

Supplementary Table S1 Food items in the quantitative and qualitative FFQs in the China Kadoorie Biobank study 2

Supplementary Table S2 Percentages of frequency levels in 12-day 24h DRs and two FFQs 3

Supplementary Table S3 Average daily intake energy of food groups in 24h DRs 4

Supplementary Table S4 Coefficients to compare the first qualitative FFQ and 12-day 24h DRs 5

Supplementary Table S5 Coefficients to compare the second qualitative FFQ and 24h DRs 6

Supplementary Table S6 Coefficients to compare the first quantitative FFQ and 24h DRs 7

Supplementary Table S7 Coefficients to compare the second quantitative FFQ and 24h DRs 8

Supplementary Figure S1 The study design to assess the relative validity and reproducibility of qualitative and quantitative FFQs in the China Kadoorie Biobank study 9

Supplementary Table S1. Food items in the quantitative and qualitative FFQs in the China Kadoorie Biobank study

	Quantitative FFQ	Qualitative FFQ
**Original groups**	1. Rice	1. Rice
	2. Wheat products	2. Wheat products
	3. Other staple foods	3. Other staple foods
	4. Meat	4. Meat
	5. Poultry	5. Poultry
	6. Fish/seafood	6. Fish/seafood
	7. Eggs	7. Eggs
	8. Fresh vegetables	8. Fresh vegetables
	9. Fresh fruit	9. Fresh fruit
**Split groups**	10. Soya products	10. Soya products (excluding liquids)
		11. Soymilk
	11. Preserved vegetables	12. Salted vegetables
		13. Pickled vegetables
	12. Dairy products	14. Milk
		15. Yoghurt
		16. Other dairy foods
**Added groups**	-	17. Dried vegetables
	-	18. Pure fruit/vegetable juice
	-	19. Carbonated soft drinks
	-	20. Other cold soft drinks

FFQ: food frequency questionnaire.

Supplementary Table S2. Percentages of frequency levels in 12-day 24h DRs and two FFQs

Food groups	24h DRs					1st qualitative FFQ					1st FFQ vs 24h DR*	2nd qualitative FFQ					2nd FFQ vs 24h DR*	1st vs 2nd FFQ*
	Never/rarely	Monthly	1-3 d/wk	4-6 d/wk	Daily	Never/rarely	Monthly	1-3 d/wk	4-6 d/wk	Daily		Never/rarely	Monthly	1-3 d/wk	4-6 d/wk	Daily		
Rice	<0.1	2.1	33.8	13.0	51.2	2.3	6.3	29.4	8.8	53.2	0.21	1.9	3.7	31.5	10.0	53.0	0.34	0.07
Wheat products	5.3	9.7	30.6	15.3	39.1	11.3	12.5	30.6	13.9	31.7	0.02	14.1	13.2	28.2	20.8	23.6	<0.05	<0.05
Other staple foods	25.2	11.3	30.8	13.2	19.4	28.5	26.6	16.0	6.0	22.9	0.01	20.4	31.7	21.5	4.4	22.0	0.04	<0.05
Meat	1.2	1.4	27.8	49.8	19.9	3.7	5.1	33.8	19.2	38.2	0.33	2.1	7.4	33.3	21.3	35.9	0.68	0.17
Poultry	31.7	22.2	43.1	3.0	<0.1	26.6	44.0	24.8	3.0	1.6	<0.05	26.4	40.5	28.0	3.5	1.6	<0.05	<0.05
Fish/seafood	34.5	15.5	38.2	10.7	1.2	37.0	25.7	28.9	5.8	2.6	<0.05	35.7	31.7	27.3	4.6	0.7	<0.05	<0.05
Eggs	9.7	6.7	49.3	27.3	6.9	10.2	13.2	34.5	13.0	29.2	0.19	7.6	9.7	33.3	20.6	28.7	<0.05	0.07
Fresh vegetables	<0.1	<0.1	<0.1	7.9	92.1	0.2	<0.1	0.2	2.6	97.0	<0.05	0.5	0.2	0.2	2.8	96.3	<0.05	<0.05
Fresh fruit	22.5	12.7	38.9	18.5	7.4	12.3	15.5	28.7	9.7	33.8	<0.05	10.9	13.0	32.2	17.4	26.6	<0.05	<0.05
Soya products	15.7	20.6	53.5	9.5	0.7	30.6	22.7	39.4	4.6	2.8	<0.05	31.3	29.4	33.1	5.1	1.2	<0.05	<0.05
Preserved vegetables	30.6	13.9	44.0	10.9	0.7	39.4	23.8	21.3	7.4	8.1	<0.05	44.7	21.8	19.4	6.2	7.9	0.11	<0.05
Dairy products	67.8	9.0	18.8	3.2	1.2	64.4	12.5	8.3	3.2	11.6	0.15	61.1	15.3	12.0	4.6	6.9	0.06	0.04

24h DR: 24-hour dietary recall; FFQ: food frequency questionnaire.* Comparisons between different three dietary assessments were tested using Wilcoxon tests.

Supplementary Table S3. Average daily intake energy of food groups in 24h DRs

Food groups	Average energy (kcal/day)	Average total energy (kcal/day)	Percentage (%)
Rice	476.1	1616.1	29.5
Wheat products	295.3	1616.1	18.3
Other staple foods	131.6	1616.1	8.1
Meat	260.8	1616.1	16.1
Poultry	28.9	1616.1	1.8
Fish/seafood	28.7	1616.1	1.8
Eggs	55.4	1616.1	3.4
Fresh vegetables	78.1	1616.1	4.8
Fresh fruit	28,0	1616.1	1.7
Soya products	33.4	1616.1	2.1
Preserved vegetables	1.6	1616.1	0.1
Dairy products	16.5	1616.1	1.0
Dried vegetables	3.7	1616.1	0.2
Carbonated soft drinks	0.2	1616.1	<0.1
Other cold soft drinks	1.1	1616.1	0.1
Foods not in FFQs	176.5	1616.1	10.9

24h DR: 24-hour dietary recall; FFQ: food frequency questionnaire. No participants consumed pure fruit or vegetable juice in the 24h DRs.

Supplementary Table S4. Coefficients to compare the first qualitative FFQ and 12-day 24h DRs

Food groups	Weighted kappa	Adjusted Spearman	Cross-classification			
			Same groups	Adjacent groups	Extreme groups	Others
Rice	0.88	0.58	75.7	19.0	0.2	5.1
Wheat products	0.80	0.36	47.7	30.8	<0.1	21.5
Other staple foods	0.80	0.24	35.9	38.4	0.9	24.8
Meat	0.60	0.31	39.6	50.0	<0.1	10.4
Poultry	0.61	0.25	35.2	46.5	<0.1	18.3
Fish/seafood	0.75	0.66	45.4	37.7	0.2	16.6
Eggs	0.67	0.54	37.7	42.1	0.5	19.7
Fresh vegetables	0.06*	-0.02*	89.6	10.0	0.2	0.2
Fresh fruit	0.71	0.50	30.6	38.0	2.5	29.0
Soya products	0.64	0.37	37.7	31.9	<0.1	30.3
Preserved vegetables	0.83	0.38	38.2	23.6	0.9	37.3
Dairy products	0.81	0.49	58.3	17.1	1.6	22.9

FFQ: food frequency questionnaire; 24h DR: 24-hour dietary recall. The weight for kappa was defined to be 1 if the frequency levels were in the same group, 0.5 if they were in adjacent groups, and 0 if they were in extreme groups. Spearman coefficients were adjusted for age, sex and region.

* Coefficients were not significant (P > 0.05).

Supplementary Table S5. Coefficients to compare the second qualitative FFQ and 24h DRs

Food groups	Weighted kappa	Adjusted Spearman	Cross-classification			
			Same groups	Adjacent groups	Extreme groups	Others
Rice	0.90	0.50	78.2	16.9	4.7	0.2
Wheat products	0.79	0.37	45.6	33.3	21.0	<0.1
Other staple foods	0.78	0.21	36.1	41.2	21.7	0.9
Meat	0.62	0.36	43.5	47.7	8.8	<0.1
Poultry	0.63	0.25	37.5	44.7	17.6	0.2
Fish/seafood	0.72	0.51	42.1	40.0	17.8	<0.1
Eggs	0.62	0.43	34.5	43.8	21.3	0.5
Fresh vegetables	0.06*	0.05*	88.9	10.2	0.4	0.5
Fresh fruit	0.73	0.55	37.7	37.5	22.9	1.9
Soya products	0.63	0.34	36.6	35.6	27.8	<0.1
Preserved vegetables	0.85	0.39	44.9	22.2	31.9	0.9
Dairy products	0.79	0.44	56.5	20.1	22.5	0.9

FFQ: food frequency questionnaire; 24h DR: 24-hour dietary recall. The weight for kappa was defined to be 1 if the frequency levels were in the same group, 0.5 if they were in adjacent groups, and 0 if they were in extreme groups. Spearman coefficients were adjusted for age, sex and region.

* Coefficients were not significant ($P > 0.05$).

Supplementary Table S6. Coefficients to compare the first quantitative FFQ and 24h DRs

Food groups	Adjusted Spearman	Weighted Kappa	Cross-classification		
			Same tertile	Adjacent tertile	Opposite tertile
**Original groups**					
Rice	0.44	0.79	67.5	32.0	0.5
Wheat products	0.36	0.66	53.1	46.6	0.2
Other staple foods	0.14	0.70	54.6	39.9	5.5
Meat	0.31	0.67	47.4	39.7	13.0
Poultry	0.30	0.67	48.3	40.6	11.1
Fish/seafood	0.45	0.72	57.2	38.9	3.8
Eggs	0.41	0.69	54.6	38.7	6.7
Fresh vegetables	0.11	0.54	36.1	46.6	17.3
Fresh fruit	0.45	0.67	52.4	42.8	4.8
**Split groups**					
Soya products (excluding liquids)	0.27	0.63	44.2	43.3	12.5
Soymilk	0.28	-	-	-	-
Salted vegetables	0.22	0.75	47.8	28.1	24.0
Pickled vegetables	0.67	-	-	-	-
Milk	0.48	-	-	-	-
Yoghurt	0.36	-	-	-	-
Other dairy foods	0.23	-	-	-	-
**Added groups**					
Dried vegetables	0.02*	-	34.6	43.5	21.9
Pure fruit/vegetable juice ǂ					
Carbonated soft drinks	0.06*	-	-	-	-
Other cold soft drinks	0.13	-	-	-	-

FFQ: food frequency questionnaire; 24h DR: 24-hour dietary recall. Original groups refer to food items shared by the qualitative and quantitative FFQ. Split groups refer to food items in the qualitative FFQ but split into subgroups in the quantitative FFQ. Added groups refer to new food items in the quantitative FFQ. The weight for kappa was defined to be 1 if the frequency levels were in the same group, 0.5 if they were in adjacent groups, and 0 if they were in extreme groups. Spearman coefficients were adjusted for age, sex and region. The blank cell indicated the percentage of zero consumption exceeded 66.7%.

* Coefficients were not significant (P > 0.05).

ǂ No participant consumed pure fruit or vegetable juice in the 24h DRs.

Supplementary Table S7. Coefficients to compare the second quantitative FFQ and 24h DRs

Food groups	Adjusted Spearman	Weighted Kappa	Cross-classification		
			Same tertile	Adjacent tertile	Opposite tertile
**Original groups**					
Rice	0.40	0.79	67.5	31.7	0.7
Wheat products	0.31	0.75	62.7	36.1	1.2
Other staple foods	0.15	0.72	53.6	37.5	8.9
Meat	0.32	0.68	48.3	38.5	13.2
Poultry	0.21	0.65	47.4	43.0	9.6
Fish/seafood	0.39	0.71	54.3	38.7	7.0
Eggs	0.41	0.69	49.3	39.7	11.1
Fresh vegetables	0.16	0.60	40.4	41.3	18.3
Fresh fruit	0.50	0.75	56.5	35.1	8.4
**Split groups**					
Soya products (excluding liquids)	0.26	0.62	44.2	41.8	13.9
Soymilk	0.26	-	-	-	-
Salted vegetables	0.38	0.86	60.1	16.8	23.1
Pickled vegetables	0.71	-	-	-	-
Milk	0.38	-	-	-	-
Yoghurt	0.35	-	-	-	-
Other dairy foods	0.39	-	-	-	-
**Added groups**					
Dried vegetables	0.06*	-	37.7	39.4	22.8
Pure fruit/vegetable juice ǂ	-	-	-	-	-
Carbonated soft drinks	0.04*	-	-	-	-
Other cold soft drinks	0.22	-	-	-	-

FFQ: food frequency questionnaire; 24h DR: 24-hour dietary recall. Original groups refer to food items shared by the qualitative and quantitative FFQ. Split groups refer to food items in the qualitative FFQ but split into subgroups in the quantitative FFQ. Added groups refer to new food items in the quantitative FFQ. The weight for kappa was defined to be 1 if the frequency levels were in the same group, 0.5 if they were in adjacent groups, and 0 if they were in extreme groups. Spearman coefficients were adjusted for age, sex and region. The blank cell indicated the percentage of zero consumption exceeded 66.7%.

* Coefficients were not significant (*P* > 0.05).

ǂ No participant consumed pure fruit or vegetable juice in the 24h DRs.

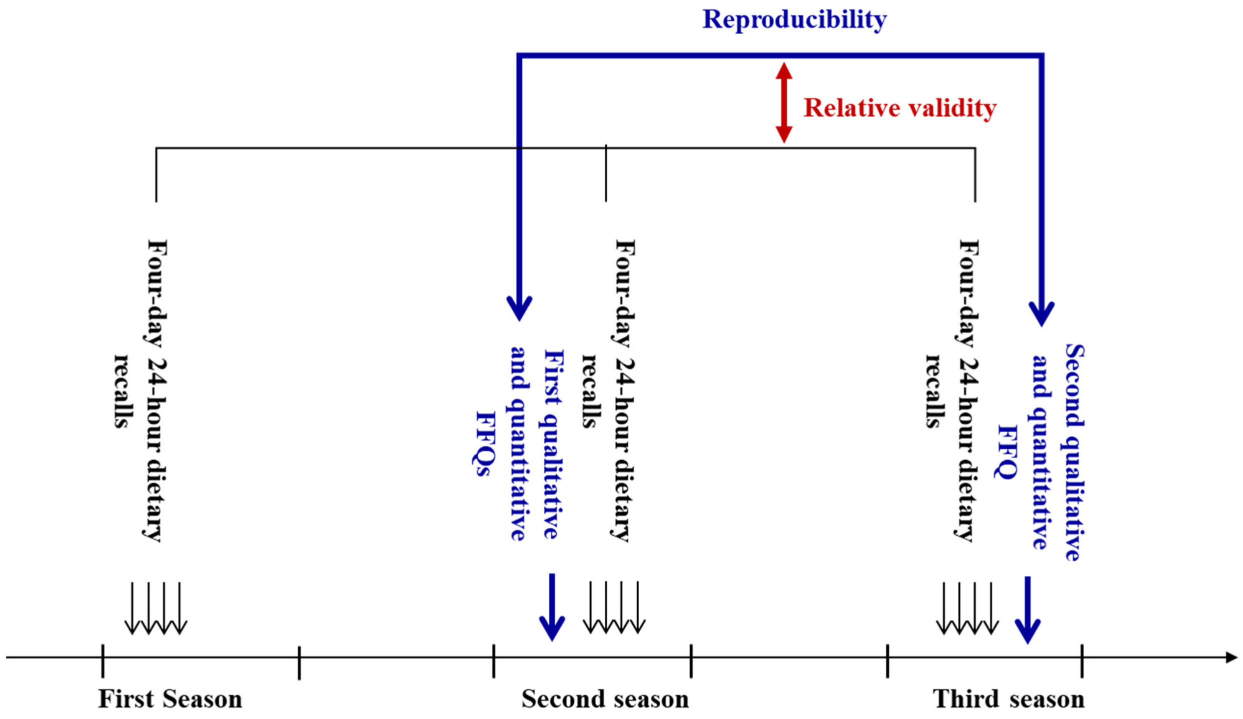

Supplementary Figure S1. The study design to assess the relative validity and reproducibility of qualitative and quantitative FFQs in the China Kadoorie Biobank study

FFQ: food frequency questionnaire.
